# Improved Fluoroquinolone-Resistant and Extensively Drug-Resistant Tuberculosis Treatment Outcomes

**DOI:** 10.1093/ofid/ofz118

**Published:** 2019-04-01

**Authors:** Eun Hye Lee, Seung Hyun Yong, Ah Young Leem, Sang Hoon Lee, Song Yee Kim, Kyung Soo Chung, Ji Ye Jung, Moo Suk Park, Young Sam Kim, Joon Chang, Young Ae Kang

**Affiliations:** Division of Pulmonology, Department of Internal Medicine, Institute of Chest Diseases, Severance Hospital, Yonsei University College of Medicine, Seoul, Republic of Korea

**Keywords:** fluoroquinolone resistance, MDR TB, treatment outcomes, XDR TB

## Abstract

**Background:**

Treatment outcomes of multidrug-resistant tuberculosis (MDR TB) remain poor, particularly for fluoroquinolone-resistant (FQ-R) MDR TB. The aim of this study was to determine treatment outcomes and factors associated with failure of MDR TB treatment, focusing on FQ resistance.

**Methods:**

Medical records were retrospectively reviewed of patients diagnosed and treated for MDR TB from January 2005 through December 2017 at Severance Hospital, South Korea.

**Results:**

Of a total of 129 patients with MDR TB, 90 (69.8%) cases were FQ-sensitive (FQ-S) and 39 (30.2%) were FQ-R. FQ-R MDR TB was associated with more severe clinical symptoms, including cavitary lesions and bilateral disease, and tended to require treatment with a greater number of drugs for a longer period of time than FQ-S MDR TB. Linezolid (51.3% vs 7.8%, *P* < .001), bedaquiline (20.5% vs 8.9%, *P* = .083), and delamanid (10.3% vs 5.6%, *P* = .452) were more frequently used in FQ-R cases. Overall, 95/124 patients (76.6%) had favorable treatment outcomes, and we did not detect a significant difference between FQ-R and FQ-S (FQ-S 65/87, 74.7%, vs FQ-R 30/37, 81.1%; *P* = .443). Old age, low body mass index, smoking, and malignancy—but not FQ resistance or extensively drug-resistant (XDR) TB—were associated with poor clinical outcomes.

**Conclusions:**

Overall, 76.6% of MDR TB patients had successful treatment outcomes. Effective drug combinations and appropriate use of new drugs may improve treatment outcomes of FQ-R MDR and XDR TB. Poor clinical outcomes were more related to the patients’ general condition rather than FQ resistance or XDR.

Multidrug-resistant tuberculosis (MDR TB) is an important public health problem, and adequate management of MDR TB is essential for effective TB control. The World Health Organization (WHO) estimated that almost 558 000 cases of rifampin-resistant TB (RR TB) occurred in 2017. Among them, 8.5% were cases of extensively drug-resistant (XDR) TB [1]. It is well known that the treatment of MDR TB is difficult due to the limitations of effective drugs, frequent adverse events, and prolonged treatment periods. With such obstacles, the global treatment success rates of MDR TB have been reported to be <70% [2, 3].

Fluoroquinolones (FQs) are considered the most important component of MDR TB treatment regimens [4]. In December 2018, the WHO released guidelines for MDR and RR TB describing key changes in recommended MDR TB treatment [5], based on a recent large-scale meta-analysis [3] and clinical trials investigating new drugs and repurposed drugs [6–8]. In those guidelines, FQ remains to be classified as a group A drug that should be recommended to all MDR TB patients. South Korea has a relatively high TB burden [9], and FQ resistance rates of 25%–30% have been reported among MDR TB patients [10]. Therefore, treatment of FQ-resistant MDR TB (including XDR TB) is a very important part of national TB control. The aim of the current study was to determine treatment outcomes and risk factors associated with treatment failure of MDR TB, focusing on FQ resistance.

## METHODS

### Study Population and Data Collection

The present study included patients who were diagnosed with MDR TB and treated at Severance Hospital, a 2500-bed university tertiary referral hospital in Seoul, South Korea, between January 2005 and December 2017. Medical records including demographic features and the results of laboratory, radiographic, and microbiological tests were retrospectively reviewed. Patients were treated by a TB specialist, and consecutive sputum cultures were performed during treatment periods according to the prescribed protocol. The research protocol was approved by the Institutional Review Board of Severance Hospital (No. 4-2018-0897). The requirement for informed consent was waived due to the retrospective nature of the analysis.

### TB Definitions

MDR TB was defined as TB resistant to isoniazid and rifampin, and XDR TB was defined as MDR TB with additional resistance to both FQ and at least 1 second-line injectable drug (SLID). Pre-XDR TB was defined as MDR TB with resistance to an FQ or SLID, but not both.

### Acid-Fast Bacillus Cultures and Drug Susceptibility Testing

Acid-fast bacillus cultures were examined by fluorochrome staining using auramine–rhodamine and culturing in 3% Ogawa medium and mycobacteria growth-indicator tube medium (MGIT; Becton Dickson, NJ, USA). MGIT has been used since 2008. Sputum cultures were repeated at least monthly until culture conversion and every 1 or 2 months thereafter. Drug susceptibility testing (DST) of *Mycobacterium tuberculosis* isolates was conducted at the Korean Institute of Tuberculosis (KIT) until December 2016, and at Seoul Clinical Laboratories (SCL) thereafter. DST was conducted at the KIT using the absolute concentration method with Lowenstein-Jensen medium [11], and SCL performed DST using agar proportion methods with Middlebrook 7H10 medium. The drugs and corresponding concentrations used are listed in Supplementary Table 1. Pyrazinamide susceptibility was determined via pyrazinamidase [12]. All patients also underwent molecular DST, and when there was a discrepancy between molecular DST and phenotypic DST, we enrolled the patient according to phenotypic DST results. In the analyses of drug effectiveness, some drugs were deemed effective if tuberculosis isolates were susceptible to those drugs. Drugs such as clofazimine, linezolid, carbapenems, bedaquiline, and delamanid were assumed to be effective, as there is or was no drug susceptibility test for those drugs [3].

### MDR TB Treatment and Treatment Outcomes

Although MDR TB treatments were individually determined by the relevant physician on a case-by-case basis, the principles of treatment were based on WHO recommendations [4, 13]. Only TB drugs used from the start of the MDR TB regimen after confirmation of DST and drugs used for at least 4 weeks were included and analyzed. According to the government policy for TB control, most MDR TB patients began treatment with hospitalization until patients were no longer considered infectious. Treatment outcomes were defined based on the revised 2013 WHO recommendations [14]. A patient was classified as “cured” if they completed treatment without treatment failure, as evident in at least 3 consecutive negative culture results at least 30 days apart after the intensive phase. “Treatment completion” was defined as a patient who had completed the treatment according to the program protocol but did not meet the definition for cured because of a lack of bacteriological results. A given treatment was deemed to have “failed” if a permanent change in treatment regimen was required due to (1) lack of conversion at the end of the intensive phase, (2) bacteriological reversion in the continuation phase after negative conversion, (3) additional acquired resistance to FQ or SLID, or (4) adverse drug responses. If patients died for any reason during the course of treatment, the outcome of these patients was designated “died.” “Lost to follow-up” was defined as treatment interruption for ≥2 consecutive months. Patients with no treatment outcome assigned were classified as “not evaluated.” If a cured patient or a patient who completed therapy resumed treatment >6 months after completion of the first treatment because of the emergence of MDR TB bacilli, the patient was classified as having a disease “relapse.” In the analysis of predictors of unfavorable outcomes, we compared treatment success (defined as cured or completion) with failure or relapse. We did not combine the outcomes of death and failure or relapse, because these cannot be considered equivalent [3] and deaths during the course of TB treatment in our study were mostly due to TB-unrelated causes. Adverse drug reactions included both abnormalities in medical examinations and subjective symptoms reported by the patients.

### Statistical Analysis

Continuous variables are presented as mean ± SD or median and interquartile range (IQR). Categorical variables are presented as number and percentage and were compared using the chi-square test or Fisher exact test. The Student *t* test and the Mann-Whitney test were used to compare continuous variables. To investigate predictors of treatment outcomes, we compared clinical variables in the treatment success and treatment failed or relapsed groups using univariate analysis. *P* values <.05 were considered statistically significant. All analyses were performed using SPSS software, version 23.0 (IBM, Armonk, NY, USA).

## RESULTS

### Baseline Patient Characteristics

Baseline characteristics of patients with FQ-S MDR TB and FQ-R MDR TB are shown in Table 1. Of the total 129 MDR TB patients, 79 (61.2%) were male, and the median age (range) was 39.7 (17–87) years. The most common comorbidity was diabetes mellitus (n = 28, 21.7%), followed by hypertension (n = 19, 14.7%), then malignancy (n = 16, 12.4%). None of the patients were infected with HIV. There were no significant differences in age, gender, underlying comorbidities, or laboratory findings between the 2 groups. Notably, however, 50/90 (55.6%) FQ-S MDR TB patients had no history of previous TB treatment, whereas only 9/39 (23.1%) FQ-R MDR TB patients revealed no previous TB treatment history (*P* = .001). Cavitary lesions and bilateral disease, as determined via chest radiography, were also more prevalent in cases of FQ-R MDR TB than in cases of FQ-S MDR TB (cavitary lesions 59.0% vs 38.9%, *P* = .035; bilateral disease 66.7% vs 47.8%, *P* = .048). With regard to drug resistance patterns, 85 patients (65.9%) had FQ-S and SLID-sensitive MDR TB, 5 (3.9%) had pre-XDR TB resistant to SLID, 28 (21.7%) had pre-XDR TB resistant to FQ, and 11 (8.5%) had XDR TB resistant to both SLID and FQ.

**Table 1.** Baseline Clinical Characteristics of 129 Patients With MDR TB

	Total (n = 129)	FQ-S MDR TB (n = 90, 69.8%)	FQ-R MDR TB (n = 39, 30.2%)	*P*
Age, y	39.7 (17–86)	38.3 (19–86)	40.0 (17–86)	.955
Male gender	79 (61.2)	54 (60.0)	25 (64.1)	.660
BMI, kg/m^2^	20.7 (13.5–41.7)	20.6 (13.5–28.7)	21.0 (16.5–41.7)	.361
Current or former smoker	54 (41.9)	36 (40.0)	18 (46.2)	.515
Comorbidities				
Diabetes	28 (21.7)	19 (21.1)	9 (23.1)	.804
HTN	19 (14.7)	16 (17.8)	3 (7.7)	.138
Chronic liver diseases	9 (7)	5 (5.6)	4 (10.3)	.452
Chronic renal failure	8 (6.2)	8 (8.9)	0	.105
Respiratory disease	7 (5.4)	6 (6.7)	1 (2.6)	.674
Malignancy	16 (12.4)	13 (14.4)	3 (7.7)	.389
Immunosuppression	2 (1.6)	2 (2.2)	0	1.0
Previous TB treatment history				
None	59 (45.7)	50 (55.6)	9 (23.1)	.001
Firstline only	47 (36.4)	35 (38.9)	12 (30.8)	.379
Second-line	23 (17.8)	5 (5.6)	18 (46.2)	<.001
Laboratory test, mean ± SD				
Hb, g/dL	13.3 ± 2.07	13.2 ± 2.14	13.6 ± 1.89	.348
Protein, g/dL	7.0 ± 0.65	7.0 ± 0.67	7.2 ± 0.56	.128
Albumin, g/dL	4.1 ± 0.57	4.1 ± 0.62	4.1 ± 0.44	.637
Cholesterol, mg/dL	161.3 ± 34.91	161.3 ± 36.71	161.3 ± 30.83	.996
Radiographic finding				
Cavity lesions	58 (45.0)	35 (38.9)	23 (59.0)	.035
Bilateral disease	69 (53.5)	43 (47.8)	26 (66.7)	.048
Positive AFB smear at treatment initiation	56 (43.4)	30 (33.3)	26 (66.7)	<.001
Drug resistance pattern				
SLID-S, FQ-S MDR	85 (65.9)	85 (94.4)	0	
FQ-S, SLID-R, MDR (pre-XDR)	5 (3.9)	5 (5.6)	0	
SLID-S, FQ-R, MDR (pre-XDR)	28 (21.7)	0	28 (71.8)	
XDR	11 (8.5)	0	11 (28.2)	

Data are presented as No. (%), mean ± SD, or median (range).

Abbreviations: AFB, acid-fast bacilli; BMI, body mass index; FQ, fluoroquinolone; HTN, hypertension; MDR TB, multidrug-resistant tuberculosis; R, resistant; S, sensitive; SLID, second-line injectable drug; TB, tuberculosis; XDR, extensively drug-resistant.

### Treatment Modalities and FQ Resistance

Table 2 shows treatment modalities according to FQ resistance. Overall, FQ-R MDR TB patients tended to be treated with greater numbers of drugs for longer periods of time, compared with FQ-S MDR TB patients (median number of drugs used, 6 vs 5; *P* = .043; median treatment duration, 24.0 vs 21.8 months; *P* = .027). Linezolid was used in 20/39 (51.3%) FQ-R MDR TB patients and 7/90 (7.8%) FQ-S MDR TB patients (*P* < .001). Bedaquiline and delamanid were also used more frequently in FQ-R patients than in FQ-S patients, but the difference was not statistically significant. Surgical resection was performed more frequently in patients with FQ-R MDR TB (11/39, 28.2%) than in those with FQ-S MDR TB (4/90, 4.4%; *P* < .001).

**Table 2.** Treatment Modalities^a^ According to Fluoroquinolone Resistance

Treatment Regimens^a^	Total MDR TB (n = 129)	FQ-S MDR TB (n = 90)	FQ-R MDR TB (n = 39)	*P*
Rifabutin	7 (5.4)	1 (1.1)	6 (15.4)	.003
Ethambutol	25 (19.4)	19 (21.1)	6 (15.4)	.450
Pyrazinamide	77 (59.7)	62 (68.9)	15 (38.5)	.001
Fluoroquinolone				
Levofloxacin	42 (32.6)	36 (40.0)	6 (15.4)	.006
Moxifloxacin	74 (57.4)	60 (66.7)	14 (35.9)	.001
Ofloxacin	1 (0.8)	0	1 (2.6)	.302
Injectable agents				
Streptomycin	21 (16.3)	15 (16.7)	6 (15.4)	.856
Kanamycin	92 (71.3)	66 (73.3)	26 (66.7)	.442
Amikacin	19 (14.7)	11 (12.2)	8 (20.5)	.222
Prothionamide	104 (80.6)	76 (84.4)	28 (71.8)	.095
Cycloserine	115 (89.1)	83 (92.2)	32 (82.1)	.122
P-aminosalicylic acid	58 (45.0)	37 (41.1)	21 (53.8)	.182
Clarithromycin	17 (13.2)	6 (6.7)	11 (28.2)	.001
Amoxicillin/clavulanate	23 (17.8)	5 (5.6)	18 (46.2)	<.001
High-dose INH	4 (3.1)	0	4 (10.3)	.007
Clofazimine	2 (1.6)	0	2 (5.1)	.090
Linezolid	27 (20.9)	7 (7.8)	20 (51.3)	<.001
Bedaquiline	16 (12.4)	8 (8.9)	8 (20.5)	.083
Delamanind	9 (7)	5 (5.6)	4 (10.3)	.452
No. of drugs used, median (range)	5 (3–9)	5 (3–9)	6 (4–9)	.043
No. of possibly effective drugs, median (range)	5 (2–7)	5 (3–7)	4 (2–7)	<.001
Duration of SLID treatment, median (IQR), mo	7.8 (4.9–9.2)	7.1 (4.4–8.3)	9.9 (6.6–15.8)	<.001
Duration of treatment, median (IQR), mo	22.6 (19.3–25.7)	21.8 (18.8–24.8)	24 (20.3–27.5)	.027
Surgical resection	15 (11.6)	4 (4.4)	11 (28.2)	<.001

Data are presented as No. (%) or median (range/interquartile range).

Abbreviations: FQ-R, fluoroquinolone-resistant; FQ-S, fluoroquinolone-sensitive; INH, isoniazid; IQR, interquartile range; MDR TB, multidrug-resistant tuberculosis; SLID, second-line injectable drug.

^a^Only TB drugs used from the start of the MDR TB regimen after confirmation of drug sensitivity testing and drugs used for at least 4 weeks were included and analyzed.

### Adverse Drug Reactions

Table 3 shows the drug-related side effects observed in the treated MDR TB patients. Regardless of FQ resistance, gastrointestinal symptoms were the most common adverse reactions, occurring in 92 of the 129 patients (71.3%). Tinnitus or hearing difficulty developed in 32/90 (35.6%) of the patients with FQ-S MDR TB and 25.6% (10/39) of the patients with FQ-R MDR (*P* = .270). Hematologic abnormalities occurred more frequently in FQ-R MDR TB patients than in FQ-R MDR TB patients (6/39, 15.4%, vs 5/90, 5.6%; *P* = .087), and peripheral neuropathy was more frequent in FQ-R MDR TB patients than in FQ-S MDR TB patients (13/39, 33.3%, vs 7/90, 7.8%; *P* < .001). These observations were possibly associated with the more frequent use of linezolid in this group [15]. Only 2 of the patients included in the study (1.5%) experienced QT prolongation. In 1 of these cases, it may have been associated with quinolone, and in the other it was associated with the use of delamanid.

**Table 3.** Adverse Drug Reactions^a^ in Patients With MDR TB

Adverse Drug Reactions^a^	Total MDR TB (n = 129)	FQ-S MDR TB (n = 90)	FQ-R MDR TB (n = 39)	*P*
Gastrointestinal trouble	92 (71.3)	66 (73.3)	26 (66.7)	.442
Musculoskeletal pain^b^	46 (35.7)	35 (38.9)	11 (28.2)	.245
Ototoxicity^c^	42 (32.6)	32 (35.6)	10 (25.6)	.270
Dermatologic abnormalities	34 (26.4)	24 (26.7)	10 (25.6)	.903
Endocrine abnormalities^d^	29 (22.5)	19 (21.1)	10 (25.6)	.571
Hepatotoxicity	21 (16.3)	15 (16.7)	6 (15.4)	.856
Peripheral neuropathy	20 (15.5)	7 (7.8)	13 (33.3)	<.001
Psychotic problems	14 (10.9)	11 (12.2)	3 (7.7)	.550
General weakness	12 (9.3)	8 (8.9)	4 (10.3)	.753
Hematologic abnormalities^e^	11 (8.5)	5 (5.6)	6 (15.4)	.087
Eye toxicity	9 (7.0)	6 (6.7)	3 (7.7)	1.0
Renal toxicity	6 (4.7)	6 (6.7)	0 (0)	.178
QT prolongation	2 (1.6)	2 (2.2)	0 (0)	1.0
Others^f^	6 (4.7)	6 (6.7)	0	.177

Data are presented as No. (%).

Abbreviations: FQ-R, fluoroquinolone-resistant; FQ-S, fluoroquinolone-sensitive; MDR TB, multidrug-resistant tuberculosis.

^a^Included any drug adverse events, not only abnormality in medical test but also patients’ subjective symptoms.

^b^Musculoskeletal pain included myalgia and joint pain, based on patients’ subjective symptoms.

^c^Tinnitus or hearing difficulty was based on subjective symptoms and auditory testing. Only newly developed symptoms after MDR TB treatment were included.

^d^Endocrine abnormalities were mainly hypothyroidism, and some hypoglycemia or hyperglycemia were also reported.

^e^Decrease of leukocyte, hemoglobin, or platelet count less than the lower normal limit.

^f^Other: fever, alopecia, anaphylactic shock.

### Treatment Outcomes

Table 4 shows treatment outcomes in 124 patients according to FQ resistance. Five patients were excluded due to ongoing treatment status. Overall, 95/124 patients (76.6%) had successful treatment outcomes, including cure (94/124, 75.8%) and treatment completed (1/124, 0.8%). FQ-R group showed longer time to sputum culture conversion (median, 1.9 vs 0.93 months; *P* = .004) and lower 6-month conversion rate than the FQ-S group (29/38, 76.3%, vs 84/86, 97.7%; *P* < .001). However, the difference of time to sputum culture conversion between the 2 groups changed over time periods (Supplementary Table 2). After active introduction of new drugs and repurposed drugs (period 2014–2017), the time to sputum culture conversion in FQ-R was shortened and showed no statistical difference between the FQ-S group and the FQ-R group. We did not detect a significant difference in the final treatment success rates between FQ-R and FQ-S MDR TB patients in the overall study periods (FQ-S MDR 65/87, 74.7%, vs FQ-R MDR 30/37, 81.1%; *P* = .443). Five patients died during the course of TB treatment. Causes of deaths were malignancy progression (3 patients), cardiac problem (1 patient), and combined pneumonia (1 patient).

**Table 4.** Treatment Outcomes in Patients With MDR TB

	Total MDR TB (n = 124)^ a^	FQ-S MDR (n = 87)	FQ-R MDR (n = 37)	*P*
Treatment success	95 (76.6)	65 (74.7)	30 (81.1)	.443
Cure	94 (75.8)	64 (73.6)	30 (81.1)	
Completion	1 (0.8)	1 (1.1)	0	
Treatment failure or relapse	11 (8.9)	8 (9.2)	3 (8.1)	1.0
Failure	9 (7.3)^b^	7 (8.0)	2 (5.4)	
Relapse	2 (1.6)	1 (1.1)	1 (2.7)	
Others^c^				
Death	5 (4.0)^d^	5 (5.7)	0	.321
Lost to FU	7 (5.6)	6 (6.9)	1 (2.7)	.673
Not evaluated	6 (4.8)	3 (3.4)	3 (8.1)	.362
Time to sputum culture conversion, mo	1.07 (0.05–2.37)	0.93 (0–1.93)	1.9 (0.64–5.93)	.004
6-mo culture conversion, No (%)	113/124 (91.1)	84/86 (97.7)	29/38 (76.3)	<.001

Data are presented as No. (%) or median (interquartile range).

Abbreviations: FQ-R, fluoroquinolone-resistant; FQ-S, fluoroquinolone-sensitive; FU, follow-up; MDR TB, multidrug-resistant tuberculosis.

^a^Analysis was performed after exclusion of 5 patients due to ongoing treatment status.

^b^Six patients had their treatment terminated earlier due to adverse drug reactions, and 3 patients were defined as failure due to lack of culture conversion by the end of the intensive phase.

^c^Excluded from further analysis.

^d^Causes of deaths were malignancy progression (3 patients), cardiac problem (1 patient), and combined pneumonia (1 patient).

### Predictors of Treatment Failure or Relapse

Comparisons between the 95 successfully treated patients and the 11 treatment failure or relapse patients are shown in Table 5. There were no statistically significant differences in gender, TB treatment history, proportion of XDR TB or FQ resistance, or laboratory findings between the 2 groups. In univariate analysis, treatment failure/relapse was associated with age (odds ratio [OR], 1.056; 95% confidence interval [CI], 1.019–1.094; *P* = .003), lower body mass index (BMI; OR, 0.732; 95% CI, 0.557–0.96; *P* = .024), history of smoking (OR, 9.29; 95% CI, 1.893–45.61; *P* = .006), and underlying malignancy (OR, 6.214; 95% CI, 1.493–25.86; *P* = .019).

**Table 5.** Clinical Characteristics Comparison According to Treatment Outcomes and Predictors of Treatment Failure or Relapse

Variables	Treatment Success (n = 95)	Failure or Relapsed (n = 11)	OR^a^ (95% CI)	*P*
Age, y	35.6 (28.8–50.1)	62.6 (47.3–73.2)	1.056 (1.019–1.094)	.003
Male gender	55 (57.9)	8 (72.7)	1.939 (0.484–7.771)	.519
BMI, kg/m^2^	21.0 (19.2–22.7)	19.9 (17.8–20.2)	0.732 (0.557–0.96)	.024
Current or former smoker	31 (32.6)	9 (81.8)	9.29 (1.893–45.61)	.006
Previous TB treatment Hx	53 (55.8)	5 (45.5)	0.66 (0.188–2.314)	.54
DM	17 (17.9)	4 (36.4)	2.622 (0.689–9.971)	.222
Chronic renal disease	4 (4.2)	1 (9.1)	2.275 (0.231–22.39)	.428
Chronic liver disease	6 (6.3)	1 (9.1)	1.483 (0.162–13.6)	.547
Malignancy	8 (8.4)	4 (36.4)	6.214 (1.493–25.86)	.021
XDR	8 (8.4)	0 (0)	0.916 (0.862–0.973)	1
FQ-R	30 (31.6)	3 (27.3)	0.813 (0.201–3.281)	1
AFB smear positive	43 (45.3)	4 (36.4)	0.691 (0.19–2.518)	.751
Cavity	45 (47.4)	3 (27.3)	0.417 (0.104–1.667)	.338
Bilateral	50 (52.6)	5 (45.5)	0.75 (0.214–2.626)	.652
Hemoglobin, g/dL	13.7 (12.4–14.7)	13.8 (13.0–14.4)	0.947 (0.698–1.283)	.724
Protein, g/dL	7.1 (6.8–7.5)	7.2 (6.7–7.6)	1.076 (0.363–6.19)	.894
Albumin, g/dL	4.2 (3.9–4.4)	4.2 (4.0–4.3)	0.626 (0.211–1.855)	.398
No. of used possible effective drugs	5 (4–5)	5 (3–5)	0.971 (0.559–1.686)	.917
Surgery	11 (11.6)	3 (27.3)	2.864 (0.66–12.43)	.159
Linezolid	19 (20.0)	4 (36.4)	2.286 (0.606–8.62)	.248
Bedaquiline	12 (12.6)	0 (0)	0.874 (0.809–0.943)	.357
Delamanid	6 (6.3)	0 (0)	0.937 (0.889–0.987)	1

Data are presented as No. (%) or median (interquartile range).

Abbreviations: AFB, acid-fast bacilli; BMI, body mass index; CI, confidence interval; DM, diabetes mellitus; FQ-R, fluoroquinolone-resistant; Hx, history; OR, odds ratio; TB, tuberculosis; XDR, extensively drug-resistant.

^a^Odds ratio for failure or relapse vs treatment success.

## Discussion

In the current study, the overall treatment success rate in MDR TB patients from 2005–2017 was 76.6%, and we did not detect a significant difference in treatment success rates of FQ-R and FQ-S patients. Factors such as older age, lower BMI, smoking, and underlying malignancy were associated with treatment failure or relapse.

FQ-R MDR TB including XDR TB has reportedly been associated with greater rates of treatment failure than FQ-S MDR TB due to an absence of effective drugs [16–19]. In a recent meta-analysis, the overall pooled successful treatment rate for FQ-S MDR TB was 62%–73% between 2009 and 2016, whereas that of FQ-R MDR TB including XDR TB was 51%–57% during the same period [3]. Recently, however, MDR/XDR TB treatment outcomes have been improved with the use of bedaquiline and delamanid [8, 20, 21], and repurposed drugs such as linezolid, clofazimine, and carbapenems also contributed to the improvement of MDR/XDR TB treatment outcomes [3, 7, 15, 22]. In the current study, FQ-R MDR TB was associated with more severe clinical symptoms such as cavitary lesions, bilateral disease, and higher acid-fast bacillus smear positivity (Table 1). Moreover, FQ-R MDR TB patients tended to receive greater numbers of drugs for longer periods of time (Table 2). However, overall numbers of adverse drug reactions did not differ significantly in patients with FQ-S and FQ-R MDR TB, and the treatment success rates were comparable, which is probably related to the more frequent use of medications such as linezolid, clofazimine, bedaquiline, and delamanid in patients with FQ-R MDR TB (Table 2). This suggests that even in cases of FQ-R MDR TB including XDR TB, improved treatment outcomes can be achieved if appropriate drug combinations are used. Several recent studies using new drugs or repurposed drugs also yielded results consistent with the present study, in which the treatment success rate of XDR TB was not inferior to that of MDR TB [21, 23–25].

In the present study, lower BMI was associated with treatment failure/relapse. This is consistent with several previous studies and a meta-analysis indicating that low BMI is a risk factor for worse outcomes and death in MDR TB and XDR TB patients [24, 26–28]. Malnutrition has well-established effects on immune function [29], and the accompanying reduction in immunity may increase susceptibility to *M. tuberculosis* [27]. Several studies have shown that smoking also increases the risk of developing TB and adversely affects baseline disease severity, bacteriological responses, treatment outcomes, and relapses in cases of drug-susceptible TB [30–33] and MDR TB [34–36]. Chronic exposure to tobacco impairs the normal clearance of secretions on bronchial mucosal surfaces and reduces the host’s defense capacity, resulting in increased susceptibility to *M. tuberculosis* [31]. Smoking also impairs the function of alveolar macrophages and reduces phagocytic ability, which is an important component of early host defense [37]. The International Union Against Tuberculosis and Lung Disease (Paris, France) emphasized the importance of smoking cessation in TB management, and recommended a programmatic approach beyond smoking cessation advice by physicians [38]. Despite the exclusion of deaths in the analysis of predictors of unfavorable outcomes, this study still showed that malignancy was associated with treatment failure/relapse. Previous studies have reported that patients with malignancy had an increased risk of developing TB and had poor treatment outcomes [39–41].

The present study suggests that improved treatment outcomes can be achieved via effective drug combinations and appropriate use of new drugs for optimal treatment durations, even in cases of FQ-R/XDR TB, and highlights an association between the patient’s general condition and poor clinical outcomes. Substantial interventions such as increased nutrition and smoking cessation via multidisciplinary approaches also need to be considered to enhance the patient’s general condition and further improve MDR TB treatment outcomes.

The limitations of the present study include its retrospective nature and relatively small sample size, which had insufficient statistical power to find a significant difference between FQ-R and FQ-S groups. Furthermore, we were unable to adjust for covariates based on the small sample size and few poor outcomes observed. As the study was also conducted at a single university hospital, the results are not necessarily representative of the overall population of South Korea. However, the majority of drug-resistant TB patients are treated at university hospitals and TB special hospitals in South Korea. Therefore, despite the main study limitations, the results of the study will contribute to a better understanding of the current state of MDR TB treatment outcomes, especially in cases of FQ-R MDR TB in South Korea.

## Conclusions

Overall, 76.6% of MDR TB patients had successful treatment outcomes in the present study, and FQ-R MDR TB and XDR TB were associated with comparable treatment outcomes. With the introduction of new and repurposed drugs, poor clinical outcomes were more related to the patient’s general condition, such as old age, low BMI, smoking, and underlying malignancy, rather than FQ resistance and XDR.

## Supplementary Data

Supplementary materials are available at *Open Forum Infectious Diseases* online. Consisting of data provided by the authors to benefit the reader, the posted materials are not copyedited and are the sole responsibility of the authors, so questions or comments should be addressed to the corresponding author.

Supplementary-Table
