# Supplementary material for: Improved Fluoroquinolone-Resistant and Extensively Drug-Resistant Tuberculosis Treatment Outcomes
Source: Open Forum Infect Dis. 2019 Apr 1;6(4):ofz118. doi: 10.1093/ofid/ofz118 (PMC6441780; doi:10.1093/ofid/ofz118)
Supplement: Supplementary-Table [file ofz118_suppl_supplementary-table.docx]

Supplementary Table 1. Drug susceptibility testing (DST) and corresponding concentrations

| Drugs | KIT (μg/mL) | SCL (μg/mL) |
| --- | --- | --- |
| Isoniazid | 0.2 | 0.2 and 1.0 |
| Rifampin | 40 | 1.0 |
| Ethambutol | 2.0 | 5.0 |
| Rifabutin | 20 | 0.5 |
| Streptomycin | 10 | 2.0 |
| Amikacin | 40 | 4.0 |
| Kanamycin | 40 | 5.0 |
| Capreomycin | 40 | 10 |
| Ofloxacin | 2.0 | 2.0 |
| Levofloxacin | 2.0 | 1.0 |
| Moxifloxacin | 2.0 | 0.5 |
| Prothionamide | 40 | 5.0 |
| Cycloserine | 30 | 30 |
| Para-aminosalicylic acid | 1.0 | 2.0 |

Abbreviations: KIT, Korean Institute of Tuberculosis; SLC, Seoul Clinical Laboratories.

Supplementary Table 2. Time to sputum culture conversion during different time periods.

|  | **Total** | **FQ-S** | **FQ-R** | ***p*** |
| --- | --- | --- | --- | --- |
| Time to culture conversion  (2005–2010), median (IQR), months | **1.40 (0.57-4.57)** | **1.0 (0.22-2.01)** | **4.57 (0.9-11.38)** | ***0.024*** |
| Time to culture conversion  (2011–2013), median (IQR), months | **0.73 (0-1.9)** | **0.47 (0-1.27)** | **2.20 (1.14-6.56)** | ***0.003*** |
| Time to culture conversion  (2014–2017), median (IQR), months | **1.17 (0.10-2.27)** | **1.52 (0.13-2.27)** | **0.73 (0.0-1.93)** | ***0.442*** |

Data are presented as medians (IQR, interquartile ranges or range).
